# On the Replica of US Pulmonary Artifacts by Means of Physical Models

**DOI:** 10.3390/diagnostics11091666

**Published:** 2021-09-12

**Authors:** Marcello Demi

**Affiliations:** Department of Bioengineering, Fondazione Toscana Gabriele Monasterio, 56124 Pisa, Italy; demi@ftgm.it; Tel.: +39-050-3152618

**Keywords:** lung ultrasound, B-lines, vertical artifacts, pulmonary artifacts, physical models

## Abstract

Currently, the diagnostic value of the artefactual information provided by lung ultrasound images is widely recognized by physicians. In particular, the existence of a correlation between the visual characteristics of the vertical artifacts, which arise from the pleura line, and the genesis (pneumogenic or cardiogenic) of a pulmonary disorder is commonly accepted. Physicians distinguish vertical artifacts from vertical artifacts which extend to the bottom of the screen (B-lines) and common vertical artifacts from well-structured artifacts (modulated B-lines). However, the link between these visual characteristics and the causes which determine them is still unclear. Moreover, the distinction between short and long artifacts and the distinction between common and structured artifacts are not on/off, and their classification can be critical. In order to derive further information from the visual inspection of the vertical artifacts, the mechanisms which control the artifact formation must be identified. In this paper, the link between the visual characteristics of the vertical artifacts (the observed effect) and the distribution of the aerated spaces at the pleural level (the cause) is addressed. Plausible mechanisms are suggested and illustrated through experimental results.

## 1. Introduction

The hypothesis that ultrasound (US) vertical artifacts, which are observed on lung ultrasound images, are generated through multiple reflections between the surfaces of the aerated spaces seems logical and consolidated thanks to both theoretical knowledge and experimental results [1,2,3,4]. A distribution of aerated spaces separated by a biological medium (channels) acoustically similar to the chest wall acts as an acoustic trap. Once a US pulse reaches the pleura plane through the chest wall, it is partially reflected towards the probe and partially transmitted to the channels provided by a specific distribution of the aerated spaces which characterizes the outer lung surface. The aerated space distribution can be organized as a compact air wall, and in this case, the acoustic energy is essentially reflected back to the probe. The size of the interalveolar septa is reasonably supposed to be comparable with a capillary lumen (less than 10 microns [5]). However, in the presence of a pathology, the aerated spaces can be separated by wider interstitial channels which are made of media that are acoustically similar to the chest wall [4,5,6,7]. In this case, the pulse energy can be partially trapped and subsequently re-radiated towards the probe after multiple reflections between the separated aerated spaces, giving rise to vertical artifacts which arise from the pleura line. A similar effect has been observed with other pairs of materials such as metal immersed in water [8,9]. The imaging parameters play a fundamental role in the formation of the artifacts, and the visibility of a vertical artifact (that is, its brightness, lateral dimension, and length) depends on multiple non-orthogonal factors including the gain, the time gain compensation (TGC), and all the parameters that can be easily set by the operator from the scanner keyboard [10,11,12]. Therefore, given the intrinsic variability of the artifacts as a function of multiple non-independent factors, including the human factor, making an objective diagnosis on the basis of the artefactual information is a difficult task. 

Despite this, the diagnostic value of the artefactual information provided by lung ultrasound images is currently widely recognized by physicians [13,14,15], and yet it is the information regarding the vertical artifacts that attracts their attention most. The existence of a correlation between the visual characteristics of the vertical artifacts, which arise from the pleura line, and the genesis (pneumogenic or cardiogenic) of a pulmonary disorder is commonly accepted [16,17,18]. Moreover, it has been observed that the structure and visibility of the vertical artifacts change when varying the pulse central frequency and that this variation can be used to formulate hypotheses on the nature of the pulmonary disease [4,19,20]. Physicians distinguish vertical artifacts from vertical artifacts which extend to the bottom of the screen (B-lines) [15,20,21] and B-lines from well-structured artifacts (modulated B-lines) [7,22]. However, the link between these visual characteristics and the causes which determine them is still unclear. Moreover, the distinction between short and long artifacts and the distinction between common and structured artifacts are not on/off, and their classification can be critical. An effective preliminary diagnosis, based only on the visual inspection of the vertical artifact, is probably possible. However, the mechanisms which control the artifact formation must be identified and rationally used to convert the artefactual information into anatomic information. In the following sections, the link between the visual characteristics of the vertical artifacts (the observed effect) and the distribution of the aerated spaces at the pleural level (the cause) is addressed. Single isolated acoustic traps connected to a lung disorder are introduced and analysed in order to provide a physical explanation for the different forms of vertical artifacts that are usually observed on US lung images.

## 2. Materials and Methods

Normal aerated lungs are characterized by a distribution of aerated spaces separated by thin interstitial spaces, and in this case, the US pulses are mostly reflected back to the probe by the pleura plane. However, in the presence of pulmonary pathologies, the thickness of the interstitial spaces (for example inter-alveolar and inter-lobular septa) increases, and the US pulses are partially transmitted to the lung surface underneath the pleura through these interstitial channels. In this case, the spatial arrangement of the interstitial spaces can give rise to acoustic traps where the pulse energy can be partially trapped among the aerated spaces. Subsequently, the slow re-radiation of the trapped energy gives rise to the vertical artifacts which are observed on lung images. The spatial configuration of the interstitial and aerated spaces can vary a lot, and many types of acoustic traps can exist, so that an exhaustive description of them is impossible. Obviously, given the numerous types of potential acoustic traps, numerous types of artifacts also exist. Vertical artifacts appear on lung images every time the thickness of the interstitial medium (tissue, blood, water), which separates the aerated spaces, increases over a certain threshold. However, a typical isolated acoustic trap, providing an isolated vertical artifact, can be considered and analysed as a source of important information on the genesis of the vertical artifacts. In this paper interstitial volumes surrounded by aerated spaces and linked to the pleura plane by means of a thickened interstitial space are simulated with some physical models.

The simplest and most immediate idea was to resort to sponges soaked with different volumes of water. Indeed, several papers can be found in the literature by authors who have embarked on this path obtaining interesting results [2,23,24,25,26]. Yet, before taking into account random models, other possibilities that allow us to maintain the indisputable advantage of the experiment repeatability can be explored.

In Section 3, a distribution of air cylinders in agar gel is analysed. The idea is to drill corresponding holes on two opposite walls of a box, to insert metallic cylinders in the holes so that they can lie between the two opposite walls of the box, to fill the box with a 3% hot solution of agar in water, to wait until the solution acquires its natural consistency (very similar to that of soft tissues) by means of the cooling process, and then to remove the metallic cylinders [27]. The result is that of placing cylindrical air spaces (mimicking the alveoli) in a material that simulates the connective tissue of the lung. Boxes of this type can be easily made by distributing metallic cylinders appropriately in order to simulate various acoustic traps.

**Figure 1:**
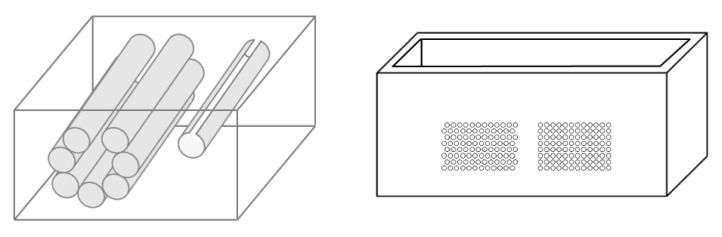
The image on the left shows a draft of an empty box with eight metallic cylinders lying between two opposite sides of the box. The image on the right shows a PVC box where two sets of 1.1 mm holes with a distance of 1.5 mm between their axes have been made using a CNC milling machine.

On the left of Figure 1, the draft of an empty box with a set of eight metallic cylinders lying between the two opposite sides of the box is shown. Seven 5.1 mm holes and an isolated 5.1 mm hole have been drilled on the two opposite sides of a simple wooden box. While the diameter of the holes was 5.1 mm, seven brass rods and one copper tube with a diameter equal to 5 mm were used to make their positioning easier. The seven holes are practically next to each other, except for the two holes at the top, which are separated by about 1 mm. The seven holes, which give rise to seven air cylinders in agar gel by means of the procedure described above, form a classic acoustic trap which is accessible from the aperture between the two air cylinders at the top. A strip of material was removed with a 2 mm diameter mill from the upper part of the copper tube and was subsequently inserted in the isolated 5.1 mm hole. In this case, once the agar solution cooled and the seven brass rods and the copper tube were pushed out, an agar volume (similar to a cylinder) surrounded by seven air cylinders was obtained on the left side of the box, while on the right side, an agar cylinder with a diameter of about 4.5 mm was obtained (the internal diameter of the copper tube). The latter was attached to the rest of the surrounding agar gel by a 2 mm wide and 0.25 mm thick peduncle surrounded by a 0.25 mm thin layer (the wall thickness of the copper tube was equal to 0.25 mm) of air for the remaining part of its surface.

Due to the significant results obtained with the above phantom, different distributions of air cylinders in agar gel were also simulated by means of a PVC box where two sets of holes had been made using a CNC milling machine. The image on the right in Figure 1 shows the PVC box with two sets of 1.1 mm holes with a distance of 1.5 mm between their axes. In this case, numerous acoustic traps can be simulated by using the same procedure: appropriate distributions of brass rods with a diameter equal to 1 mm are introduced in the holes, the box is subsequently filled with a hot solution of agar in water, and at the end of the cooling process, the brass rods are removed.

The results obtained with these first phantoms led us to develop a device which allowed us to simulate two particular acoustic traps completely surrounded by air: (i) a volume of fluid linked to the chest wall by means of a thickened interalveolar space and (ii) an interlobular septum.

**Figure 2:**
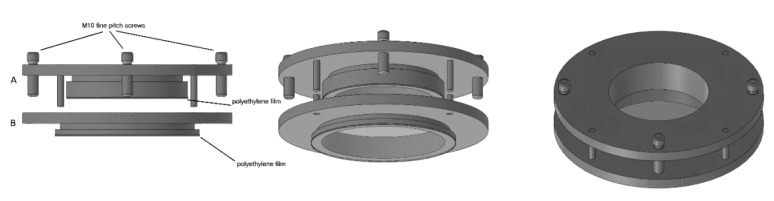
The figure shows a drawing of the device with the two membranes which seal the bottom of two containers A and B and the way it is assembled.

The device is based on two membranes separated by an adjustable air thickness. The two membranes seal the bottom of two cylindrical PVC containers (A and B) which were shaped by means of a lathe. The external diameter of the A container is 2 mm smaller than the internal diameter of the B container, so that the A container can slide inside container B. In so doing, the air thickness between the two membranes, which seal the bottom of the two containers, can vary. The membrane which seals the bottom of the B container (the lower membrane in Figure 2) acts as a simple support of the acoustic trap while the membrane which seals the bottom of the A container (the upper membrane in Figure 2) simulates the pleura. The two membranes were obtained from two simple polyethylene films which are commonly used for food packaging, and they are secured by means of two O-rings at the throat, which is visible in Figure 2 under the heads of the containers. The A container is filled with 20 mm of purified water in order to simulate the chest wall and to provide an excellent acoustic matching with the probe. The diameter of the top aperture of container A is larger than the head of the probe and makes interfacing the probe and the purified water simple. The acoustic trap under examination (a sample of agar gel) is placed on the lower membrane. Three micrometric M10 fine pitch screws (1 mm for each turn of the screws) fine-tune the air thickness between the two membranes and allow the upper membrane to come close to the acoustic trap in order to ensure an input channel to the ultrasound beam transmitted by the probe through the purified water. Figure 2 shows a drawing of the device with the two membranes which seal the bottom of the two containers A and B and the way it is assembled.

**Figure 3:**
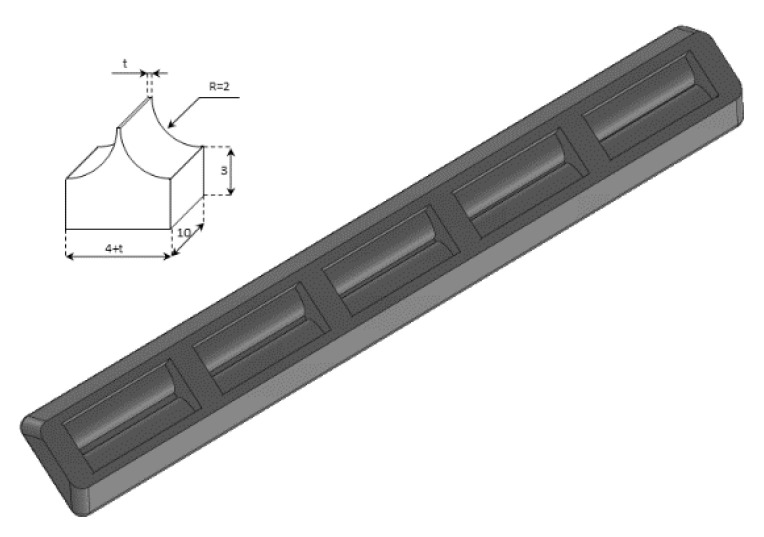
A graphic representation of the agar models with a cusp shape and a drawing of the moulds obtained with the 3D printer are shown.

A phantom in the shape of a cusp was chosen to simulate a volume of fluid linked to the chest wall by means of a thickened interstitial space. A 3% hot solution of agar was poured into some moulds which were obtained with a 3D printer, and, once cooled, the agar models were removed from the moulds and checked under a magnifying glass with a digital calliper. Agar cusps such as the one illustrated in Figure 3 with a thickness *t* varying between 0.1 and 2 mm were obtained. The agar cusps thus obtained are *3* × *(4 + t)* × *10* mm parallelepipeds with the upper surface which is modelled as a 2 mm high cusp. The agar samples were then placed between the two polyethylene films, taking care to limit the contact of the upper film to the ridge of the agar cusps.

Another mould was prepared with the 3D printer to simulate an interlobular septum. The aim was to place a micrometric agar septum between the two polyethylene films. The objective is not simple, but it is possible by using two pairs of moulds. The internal pair of moulds is the support for the agar septum. They are separated by means of two washers and assembled by means of two screws which are never removed. The external pair of moulds is pressed against the internal pair of moulds in order to form a thin septum in their central part. Two calibrated washers limit the minimum distance between the pair of external moulds and set the thickness of the septum. The two pairs of moulds so assembled are subsequently placed in a box that will be filled with an agar solution in hot water. Once the agar solution has assumed its elastic consistency, the assembled pieces are extracted from the box, and the pair of external moulds are gently removed. In so doing the pair of internal moulds are now separated by a calibrated agar septum and can be placed between the two polyethylene films of containers A and B. Here, again, the obtained septa were checked with a digital calliper under a magnifying glass.

**Figure 4:**
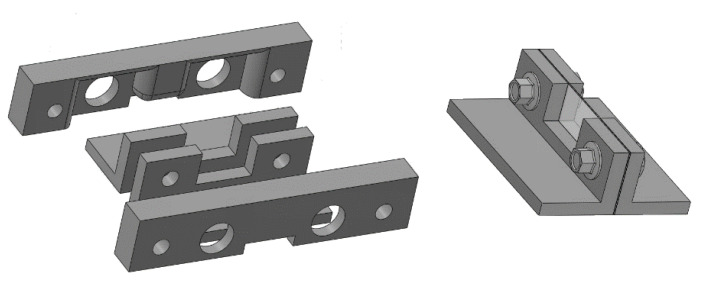
The figure shows a drawing of the two pairs of moulds, the way they are assembled, and the agar model of interlobular septum in its support.

Figure 4 shows a drawing of the two pairs of moulds, the way they are assembled, and the agar model of interlobular septum in its support (the pair of internal moulds), which is placed between the two polyethylene films of containers A and B. These pairs of moulds were printed by setting the spatial resolution of the 3D printer to 0.1 mm (the best resolution of our 3D printer). However, in order to simulate a “footprint” that the alveoli can introduce on the walls of the interlobular septa, a second pair of external moulds was also printed by setting the spatial resolution of the 3D printer to 0.280 mm. In so doing, both agar septa with smoother surfaces and agar septa with rough surfaces were obtained, and their response to an ultrasound pulse was analysed.

The US phantom images were acquired with a research programmable US scanner, the UlaOp scanner [28] developed by the University of Florence. All images were acquired by using an ultrasound pulse with a 6 MHz central frequency and a duration equal to 3 cycles. A Hanning window was also used as a temporal apodization function, and, in so doing, pulses with a bandwidth of 5.4 MHz at −12 db were obtained. The 6 MHz central frequency was chosen since this is the frequency which in our experience better highlights the modulated B-lines often observed in cardiogenic lung edema [4,7,22]. The maximum amplitude of the pulse allowed by the scanner was used, and the focus position was set at the apertures of the acoustic traps. A 192-element linear probe LA533 from Esaote was used, and 64 elements of the probe were used to transmit the pulses and to receive the echo signals. A Hanning window was also used as a spatial apodization function both in transmission and in reception. In order to reconstruct the images, a propagation speed of 1500 m/s was assumed, and the I- and Q-components of the demodulated RF signals were filtered with low pass filters with a bandwidth of 2.7 MHz at −12 db. The probe was clamped onto an XYZ linear axes electronic positioning system manufactured by IEF-Werner GmbH.

## 3. Results

### 3.1. Two Simple Models

**Figure 5:**
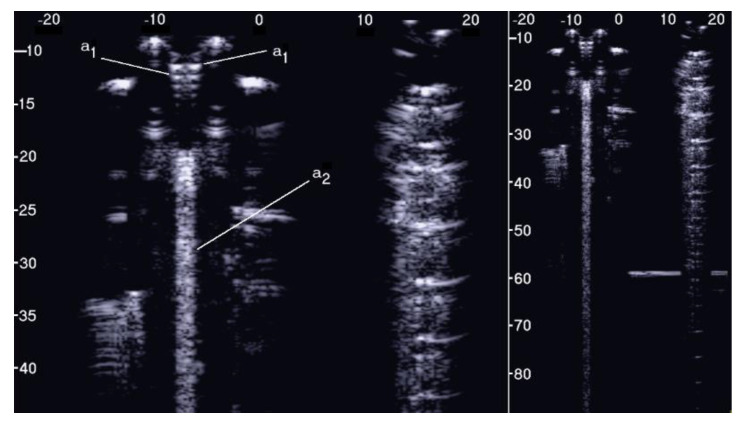
Two ultrasound vertical artifacts with different zoom degrees, which were obtained on the models illustrated in the left image of Figure 1, are shown. The pair of short artifacts a_1_ is provided by multiple reflections between the two air cylinders which limit the aperture of the trap. The longer artifact a_2_ is generated by the re-radiation of the acoustic energy and starts when the beam is reflected from the bottom of the trap.

Figure 5 shows the two ultrasound vertical artifacts with different zoom degrees, which were obtained on the models that are illustrated in the left image of Figure 1. The seven air cylinders provide two different types of artifacts (a_1_ and a_2_) which are indicated in Figure 5. The pair of short artifacts a_1_ is provided by multiple reflections between the two air cylinders which limit the aperture of the trap. In fact, a distance of about 1 mm between two replicas of their repetitive patterns can be derived from Figure 5 according to the assumed propagation speed of 1500 m/s. The longer artifact a_2_ is generated by the re-radiation of the acoustic energy which has been transmitted to the trap and starts when the beam is reflected from the bottom of the trap. The artifact generated by the agar cylinder has a repetitive pattern that is not seen in the artifact generated by the seven air cylinders and seems to be related to the diameter of the trap. In fact, a distance of about 5 mm between two replicas of the repetitive pattern can be derived from Figure 5 according to the assumed propagation speed of 1500 m/s. This simple example shows how different artifacts can be obtained from acoustic traps with similar volumes and similar shapes. This example also shows how long artifacts can be obtained when an interstitial volume, surrounded by aerated spaces, is linked to the pleura plane by means of a small (as compared to the interstitial volume) channel.

### 3.2. A Distribution of Air Cylinders in Agar Gel

**Figure 6:**
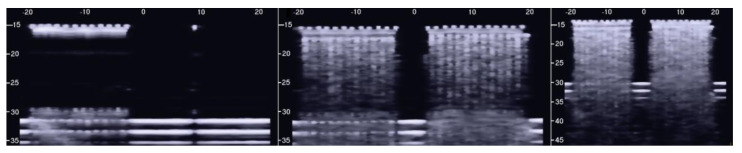
The image on the left shows how a single row of air cylinders does not generate vertical artifacts. The two images in the centre and on the right show the artifacts obtained with two and with four rows of air cylinders. A different zoom degree has been used for the image on the right.

The PVC box, which is shown in the right image of Figure 1, was designed to test the ultrasound response to different distributions of air cylinders. The first test shows what happens when a single row of air cylinders is introduced in the agar gel. The image on the left of Figure 6 shows that in this case, there are no artifacts except the short ones which are generated by multiple reflections between two contiguous air cylinders. In fact, a distance of about 0.5 mm between two replicas of the repetitive pattern can be derived from Figure 6 according to the assumed propagation speed of 1500 m/s. In particular, no artifact is generated by a single air cylinder. The situation changes, however, as a row of air cylinders is added, and increasingly longer artifacts are observed as the number of rows of cylinders increases. The two images in the centre and on the right of Figure 6 show the artifacts which were obtained with two and with four rows of air cylinders, respectively. A repetitive pattern is still perceivable, but it is not clearly quantifiable. The thick white lines at the bottom of the three images are given by the reverberations within the bottom wall of the box.

**Figure 7:**
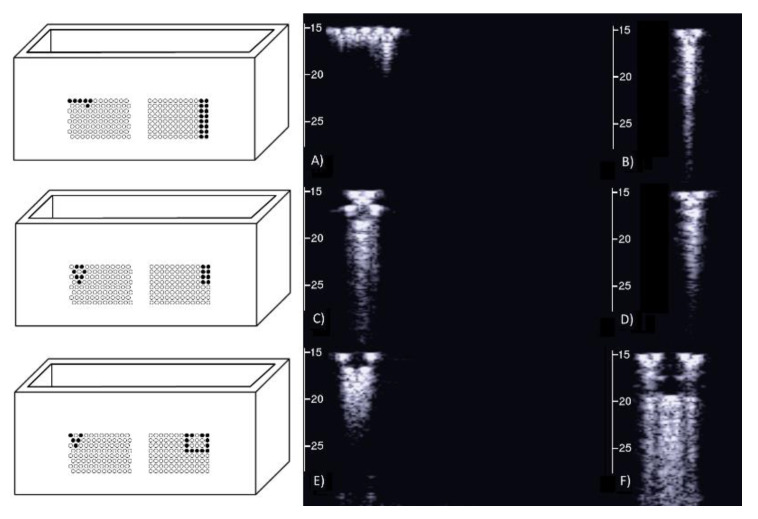
Six different arrangements of air cylinders immersed in agar gel and the obtained artifacts are shown. The black dots indicate the position of the air cylinders.

However, the box allows the examination of numerous other configurations. Figure 7 shows some configurations which were tested and the obtained artifacts. Here, it can be observed how traps with the same input channel provide different artifacts as the shape of the trap changes. Artifact A was obtained with three staggered air cylinders. The artifact is short, and it is the only one that shows the characteristic modulation of cardiogenic artifacts [7,22]. The modulation is most likely related to the simplicity of the trap which favours the constructive sum of the echoes, while its length is not easy to interpret. Artifacts B and D are generated by configurations of air cylinders which form two channels with rough lateral surfaces and the different lengths suggest their probable origin. The two artifacts are probably generated by multiple reflections between the walls of the channels during the propagation of the acoustic wave from the top to the bottom of the two channels. Artifact C is generated by a configuration of cylinders similar to that illustrated on the left of Figure 1 with cylinders of a larger diameter. Here, it can be observed how a larger trap with a more complex internal geometry than trap A provides a longer and more confused artifact. Artifact E was generated by a configuration of air cylinders obtained by eliminating the two cylinders that form the input channel of the trap that generated artifact C. This example shows how very different artifacts can be obtained when varying the shape and the size of the access channel. The F artifact is particular since in this case the US energy can be transmitted to the trap through the two small (0.5 mm large) lateral channels and through the larger (2 mm large) central channel. In this case, the artefactual information can be seen as three close but different artifacts or as a single complex artifact.

The above tests on the PVC box provided important indications: first of all, the confirmation that the artifacts observed on the ultrasound pulmonary images are probably generated by multiple reflections between the aerated spaces, since the images acquired on the box in the presence of a single air cylinder did not show any artifacts. The second indication is that traps with the same input channel can provide different artifacts as the shape of the trap changes. The visual characteristics of a vertical artifact depend on the trap shape and on the input channel. Moreover, tests on simulated single traps did not give rise to artifacts of significant length. Only in the case of the artifact in Figure 5, which was generated by the seven air cylinders, was an artifact of 12 cm observed. Even though the vertical artifacts cannot be objectively split into short and long artifacts, given the great emphasis placed by physicians on their observation regarding artifacts which extend to the bottom of the screen, such a strong indication cannot be neglected. Besides the length of the artifacts, however, another problem arose from the previous tests: none of the artifacts obtained with the air cylinders in agar gel showed those clearly modulated artifacts which are often observed in cardiogenic patients [7,22].

### 3.3. Agar Gel Samples Completely Surrounded by Air

**Figure 8:**
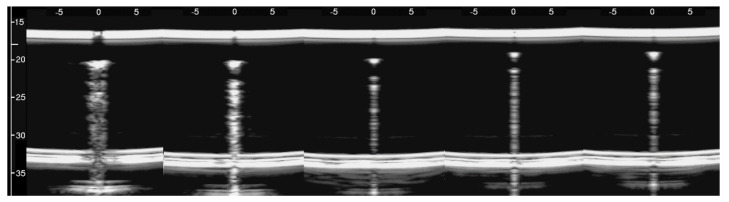
From left to right, the figure shows the vertical artifacts which were obtained with models of agar cusps having a thickness t of the upper part equal to 2, 1, 0.5, 0.3, and 0.1 mm, respectively. The two thick white lines at the top and at the bottom of the images represent the reflection of the upper polyethylene film and its replica, respectively.

Figure 8, from left to right, shows the details of the artifacts which were obtained on agar cusps with a thickness *t* equal to 2, 1, 0.5, 0.3, and 0.1 mm, respectively (see Figure 3). A distance of about 4 mm, between the upper polyethylene film and the first reflection generated by the agar cusps, is derived from the figure, according to the assumed propagation speed of 1500 m/s. This means that the US pulse, once it has been transmitted to the ridge of the agar cusps, propagates through the agar sample and reaches the lower polyethylene film before being reflected towards the probe. Figure 8 shows how modulated B-lines are obtained when the thickness *t* of the coupling section is equal to 0.5, 0.3, and 0.1 mm, and how confused artifacts are obtained when the latter increases. A slightly confused modulation was obtained when the thickness of the cusp ridge was equal to 1 mm. Modulated artifacts, such as those observed in cardiogenic pathologies, are finally obtained for the first time on deterministic phantoms, and they seem to be correlated to the size of the acoustic channel which links the acoustic trap to the chest wall.

**Figure 9:**
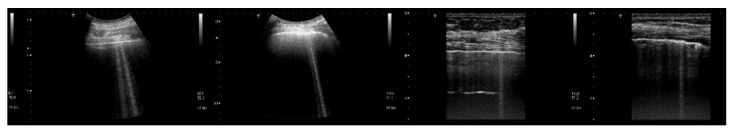
From left to right, the first image shows two B-lines; the first B-line does not show any modulation, while the second shows a slightly confused modulation. The second image shows a modulated B-line, and the third shows a non-modulated B-line. The last image on the right shows a modulated B-line.

Figure 9 shows four lung US images which have been selected from a data set previously acquired by means of a Toshiba Aplio XV scanner in our Respiratory Department during a study which was approved by the local Ethics Committee CEAVNO (study number 1089 approved on January 30, 2017). The two images on the left were acquired with a PVT-375BT convex probe and a central frequency equal to 6 MHz. The two images on the right were acquired with a PLT-704AT linear probe and a central frequency of 7.2 MHz. From left to right, the first image shows two B-lines; the first B-line does not show any modulation, while the second shows a slightly confused modulation. The second image shows a modulated B-line and the third shows a non-modulated B-line. The last image on the right shows a modulated B-line. An analogy with the experimental results illustrated in Figure 8 emerges, and the physicians’ hypothesis regarding the progression of a lung disorder supports this thesis. According to their hypothesis, the interstitial spaces between the alveoli gradually increase with the progression of a pathology, and the modulated B-lines are related only to the early stages of the pathology. 

### 3.4. Simulated Interlobular Septa

**Figure 10:**
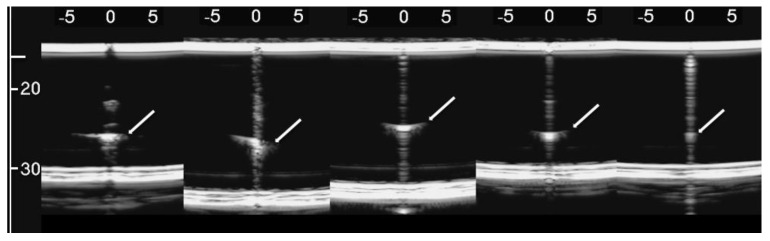
From left to right: the first four images show the vertical artifacts which were obtained with agar septa having smoother lateral surfaces and thickness equal to 1.2, 0.8, 0.6, and 0.4 mm. The last image on the right shows the vertical artifact which was obtained with an agar septum with rough lateral surfaces and a thickness equal to 0.4 mm. The two thick white lines at the top and at the bottom of every image represent the reflection of the upper polyethylene film and its replica, respectively. The arrows indicate the reflection of the lower polyethylene film.

The simulated septa, which were obtained by using the pair of external moulds printed by setting the spatial resolution of the 3D printer to 0.1 mm, were first analysed. The first four images in Figure 10 from left to right show the artifacts which were obtained on four different simulated septa with smooth lateral surfaces and with thicknesses equal to 1.2, 0.8, 0.6, and 0.4 mm. It is worth noting that in this case, the vertical artifacts begin just below the upper polyethylene film. The septa with a thickness equal to 0.8, 0.6, and 0.4 mm provided modulated artifacts even though the 0.8 mm septum showed a slightly confused modulation. However, Figure 10 also shows an unexpected acoustic sign which is not observed on US lung images. The whiter sign, which is observed within the modulation of the three artifacts, is given by the reflection of the lower polyethylene film. The bottom of the septa gives rise to a reflection which is clearly highlighted in Figure 10, and this experimental result is not consistent with the observations of the physicians. Therefore, the simulated septa, which were obtained by using the pair of external moulds printed by setting the spatial resolution of the 3D printer to 0.280 mm, were also analysed. The vertical artifact which was obtained on a 0.4 mm septum with rough lateral surfaces is shown in the last image on the right of Figure 10. The strong reflection of the bottom of the septum is still perceivable, but this time, it is partially masked within the vertical artifact, and the latter is closer to the artifacts which are observed on US pulmonary images. Modulated vertical artifacts were obtained, and a correlation with the thickness of the septa emerges again, as expected by physicians.

## 4. Discussion

Some deterministic models have been introduced in this paper in order to replicate the visual characteristics of the vertical artifacts which are often observed on lung US images in the presence of pathologies. In this phase, deterministic models were preferred in order to guarantee the experiment repeatability. The device illustrated in Figure 2 combined with the agar phantoms and the moulds provided by a 3D printer proved to be an interesting tool, and the technique illustrated in the image on the right of Figure 1 with the PVC box can be further developed. For example, the CNC milling machine can be replaced by laser technology and PVC boxes with aerated space distributions closer to the pulmonary anatomy (smaller holes having smaller distances among their axes) can be obtained. The model of multiple air cylinders arranged in multiple overlapping rows simulates a cloud of alveoli separated by large interstitial spaces which is perhaps what happens when observing the so-called white lung artifact [4]. These experimental models can represent the basis for numerous quantitative studies on vertical artifacts.

In a previous paper [4], the term acoustic trap was introduced. Here, it is illustrated by means of numerical models how a single trap, given by a volume of tissue mimicking medium surrounded by air, can produce a modulated vertical artifact and how its modulation is correlated with the shape and the size of the trap. In this paper, the potential role of the interstitial channel, which allows both the transmission of the acoustic energy of the pulse to the trap and the gradual re-radiation of the trapped energy towards the probe, is highlighted by means of physical experimental models. Modulated artifacts, such as those observed in cardiogenic pathologies, are obtained for the first time on deterministic physical phantoms.

The vertical artifacts which are observed in LUS images have some peculiarities that are probably strictly correlated with important anatomical pathological characteristics of the observed organ and are worth analysing. The length of an isolated vertical artifact is an example. In our experience the artifacts “which start at the pleura line and extend to the bottom of the screen” (the so-called B-lines) are obtained if the trap volume is completely surrounded by air except for a small input/output channel, so that the trapped energy cannot easily leave the trap as in the case of the artifact which is provided by the seven air cylinders (Figure 5). The structure of an isolated artifact is another example. The modulated artifacts were observed on cusps and septa when their link to the polyethylene film of container A was limited to a small acoustic channel. The characteristic modulation of the artifacts gradually disappears when increasing the section of the acoustic channels as shown in Figure 8 and Figure 10. Here, a hypothesis, which should be analysed, can be formulated: when the link between the trap and the polyethylene film is reduced to a small acoustic channel, the trap acts as a point-like source of ultrasound and, consequently, can both re-radiate the trapped energy slowly and eliminate the uneven acoustic perturbation of the particles of the medium at the top of the channel. However, the analysis of this result from a clinical point of view is most important since it seems to match with a physician’s focal hypothesis. Physicians relate the presence of modulated B-lines with an early stage of the cardiogenic pulmonary edema or pulmonary inflammation, while they relate the presence of simple B-lines with advanced stages of the two pathologies when the interlobular septa and the interstitial spaces between contiguous alveoli are further enlarged [7,22,29,30]. The experimental results obtained both on simulated interalveolar spaces and interlobular septa provide a rational explanation for the different forms of vertical artifacts that are usually observed on US lung images. The B-line modulation changes and becomes progressively more confused until the artifacts assume a structure which is visually similar to speckle noise in advanced stages of a pathology when the lung density increases. 

## 5. Conclusions

The device illustrated in Figure 2 combined with the moulds provided by a 3D printer and the PVC box are versatile tools and can be used to check models of the outer lung surface that physicians may suggest on the basis of their anatomopathological knowledge. The geometry of the models can also be progressively modified to mimic various degrees of severity of a pathology, as in the case of the cusps and the septa which have been analysed here when varying the size of their link with the water container. The visual inspection of the artifacts obtained on these models can be a helpful guide for physicians in their interpretation of the clinical data. What is probably most important, however, is the indication provided by the models on the informative content of the visual characteristics of the artefactual information, i.e., an indication of their capability to distinguish two pathologies and estimate their severity. This is another important aspect of the introduced tools; they can be used to investigate the sensitivity of the artifact visual inspection when varying the severity level of a simulated pathology. For example, looking at images in Figure 8, a clear difference among the modulated artifacts obtained when the thickness of the agar cusps varies from 0.5 mm to 0.1 mm does not emerge. According to the experimental results, the visual inspection of these artifacts alone does not allow a physician to distinguish an inter-alveolar space of 0.5 mm (the size of the trap input channel) from an inter-alveolar space of 0.1 mm.

The experimental results obtained on the agar cusps and on the simulated interlobular septa support the physicians’ hypothesis, according to which the modulated artifacts are related to the first stages of a pathology, while the presence of simple B-lines is related to advanced stages of the pathology when the interlobular septa and the interstitial spaces between contiguous alveoli are further enlarged.

The experimental results also justify the particular interest of physicians concerning the artifacts which extend to the bottom of the screen, since in our experience, these artifacts can only be provided by particular traps. Very long artifacts are obtained if the trap volume is completely surrounded by air except for a small input/output channel, so that the trapped energy cannot easily leave the trap.

However, the length of a vertical artifact also depends on many imaging parameters (pulse central frequency and bandwidth, pulse amplitude, attenuation, and time gain compensation, just to mention a few), and, consequently, even the same acoustic trap can generate artifacts with different lengths. The artifact length alone does not characterize the trap, and splitting the vertical artifacts into short and long artifacts can be misleading.

Physicians should also carefully consider another experimental result illustrated in this paper. Modulated B-lines have been obtained even with acoustic traps (Figure 3 introduces cusps with a body of 3 mm × 4 mm × 10 mm), which are probably much larger than those they expect to find in patients with cardiogenic edema.

The most important problem of the techniques illustrated in this paper is represented by the perishability of the obtained phantoms, which limits their use for the training of physicians. Durable phantoms must be developed for this purpose in the future by professional manufacturers. However, the size of the models is another problem. Agar cusps with a body of 3 mm × 4 mm × 10 mm have been used to obtain the results illustrated in Figure 8. Smaller acoustic traps similar to a real pulmonary trap cannot be analysed with this technique. At this stage of our work, a 3D printer is used to print the moulds which are subsequently used to obtain the agar models. Printing the models directly with a 3D printer would be an important step forward, and we are currently working in this direction. A tissue mimicking material with appropriate acoustic properties which is also suitable for feeding a 3D printer is needed.
